# A transcriptomic approach highlights induction of secondary metabolism in citrus fruit in response to *Penicillium digitatum *infection

**DOI:** 10.1186/1471-2229-10-194

**Published:** 2010-08-31

**Authors:** Luis González-Candelas, Santiago Alamar, Paloma Sánchez-Torres, Lorenzo Zacarías, Jose F Marcos

**Affiliations:** 1 Departamento de Ciencia de los Alimentos, Instituto de Agroquímica y Tecnología de Alimentos (IATA-CSIC), Apartado de Correos 73, Burjassot, E46100-Valencia, Spain; 2 Insituto Valenciano de Investigaciones Agrarias, Carretera Moncada - Náquera, Km. 4,5. Moncada, E46113-Valencia, Spain

## Abstract

**Background:**

Postharvest losses of citrus fruit due to green mold decay, caused by the fungus *Penicillium digitaum*, have a considerable economic impact. However, little is known about the molecular processes underlying the response of citrus fruit to *P. digitatum*.

**Results:**

Here we describe the construction of a subtracted cDNA library enriched in citrus genes preferentially expressed in response to pathogen infection followed by cDNA macroarray hybridization to investigate gene expression during the early stages of colonization of the fruit's peel by *P. digitatum*. Sequence annotation of clones from the subtracted cDNA library revealed that induction of secondary and amino acid metabolisms constitutes the major response of citrus fruits to *P. digitatum *infection. Macroarray hybridization analysis was conducted with RNA from either control, wounded, ethylene treated or *P. digitatum *infected fruit. Results indicate an extensive overlap in the response triggered by the three treatments, but also demonstrated specific patterns of gene expression in response to each stimulus. Collectively our data indicate a significant presence of isoprenoid, alkaloid and phenylpropanoid biosynthetic genes in the transcriptomic response of citrus fruits to *P. digitatum *infection. About half of the genes that are up-regulated in response to pathogen infection are also induced by ethylene, but many examples of ethylene-independent gene regulation were also found. Two notable examples of this regulation pattern are the genes showing homology to a caffeine synthase and a berberine bridge enzyme, two proteins involved in alkaloid biosynthesis, which are among the most induced genes upon *P. digitatum *infection but are not responsive to ethylene.

**Conclusions:**

This study provided the first global picture of the gene expression changes in citrus fruit in response to *P. digitatum *infection, emphasizing differences and commonalities with those triggered by wounding or exogenous ethylene treatment. Interpretation of the differentially expressed genes revealed that metabolism is redirected to the synthesis of isoprenes, alkaloids and phenylpropanoids.

## Background

Citrus is one of the most economically important fruit crops in the world. Harvested fruits are usually stored before they reach the market for fresh consumption. During this postharvest period fruits are subjected to both biotic and abiotic stress conditions. *Penicillium digitatum*, the causal agent of green mold, is the major pathogen of citrus fruit worldwide during postharvest storage. Control of this fungus is mostly based on the use of chemical fungicides, such as imazalil or thiabendazol. However, problems associated with the use of these compounds are leading to the development of new and safer control alternatives, which mostly rely on biological control microorganisms, physical or chemical treatments either as standalone or combined treatments. Another approach focuses on improving the natural defense capability of the fruit. However, despite the economic relevance of losses due to *P. digitatum *infection, there have been few studies directed to unravel citrus fruit responses to pathogen invasion or to elicitors that increase resistance against pathogen infection.

It is well known that the flavedo (outer colored part of the rind) is more resistant to *P. digitatum *than the albedo (inner white part) [1,2]. This fact has been classically associated with the presence of both preformed and induced antifungal compounds in the flavedo [3]. Moreover, the concentration of some phytoalexins, such as scoparone, increases in the flavedo in response to *P. digitatum *attack, although a much higher induction is achieved by treatments that increase resistance in the fruit [4,5]. Other responses triggered by this fungus include the induction of PR proteins, such as β-1,3-glucanase and chitinase, and phenylalanine ammonia lyase (PAL), which catalyzes the first step in the phenylpropanoid pathway [1,6-8].

Ethylene is a major modulator of many processes in plants, including regulation of defense responses to pathogen attack [9]. Increase in ethylene production by pathogen infection is a well characterized process. In citrus fruit, infection with *P. digitatum *enhances ethylene emission, which is provided by both the fruit and the fungus [10,11]. Many of the aforementioned responses of citrus fruit to *P. digitatum *infection are at least partially dependent on this hormone [12]. The relevance of ethylene in the defense response has also been shown by the increased susceptibility to the pathogen when ethylene perception was blocked by the ethylene antagonist 1-methyl cyclopropene[12,13].

Being *P. digitatum *a successful pathogen of citrus fruit, it must be able to overcome the fruit's defense barriers. Thus, besides triggering different defense responses in the host, it is also able to suppress different lines of defense. The first evidence of this suppression of defenses was the observation that ethylene-mediated induction of PAL was greatly reduced in the presence of the fungus [14]. We have previously shown that this suppression seems to involve posttranscriptional regulation because *Pal *gene induction was not accompanied by induction of enzyme activity [1]. Production of reactive oxygen species (ROS) has also been shown to be suppressed in citrus fruit inoculated with *P. digitatum*, whereas inoculation with *P. expansum*, a closely related species but non-pathogenic on citrus fruit, triggers the production of ROS at attempted penetration sites [15]. On the other hand, the activity of different enzymes involved in the metabolism of ROS decreases in *P. digitatum*-infected fruit, albeit flavedo and albedo exhibit different patterns of enzyme inactivation [1].

Construction of cDNA libraries and generation of ESTs have become useful tools to identify plant genes responsive to pathogens [16-19]. The Citrus Functional Genomics Project (CFGP, http://bioinfo.ibmcp.upv.es/genomics/cfgpDB/) aims to characterize the main biological and agronomical traits of citrus [20]. As a key part of this project, a citrus EST collection has been generated from 53 cDNA libraries covering different tissues, developmental stages and stress conditions. One of these libraries, RindPdig24, was obtained from the flavedo and albedo of citrus fruit (*C. clementina*) infected with *P. digitatum*. In order to gain a better understanding of the citrus fruit's responses to *P. digitatum *infection we sought to identify citrus genes that are up-regulated in response to this pathogen. In the present work we describe the construction and analysis of a subtractive cDNA library, named RindPdigS, enriched in defense-related mRNAs. A cDNA macroarray derived from this subtracted cDNA library has been used to interrogate the role of ethylene in the regulation of citrus genes induced by *P. digitatum *infection.

## Results

### Construction of a subtracted cDNA library enriched in pathogen-responsive ESTs

We used the SSH method [21] to dissect the response of citrus fruit to *P. digitatum *by obtaining a cDNA library (named "RindPdigS") enriched in citrus genes that have higher expression upon *P. digitatum *infection (I) than in the corresponding wounded controls (W). Infected tissue was collected 24 hours post-inoculation (hpi) to allow identification of early fruit responses and to maximize the number of fruit genes, since under these conditions the first symptoms of fruit maceration by the fungus begin at 48 hpi. We have previously demonstrated that some defense-related genes start to increase their expression at 24 hpi, but maximum expression was reached at 48 hpi [12].

A nylon filter array was constructed with PCR amplicons from 1,436 randomly picked clones from the RindPdigS library, as well as 100 positive and negative controls (see Materials and Methods). In a confirmatory experiment, the macroarray was hybridized with labelled cDNAs from the same samples used for the construction of the subtracted library. As a result of the analysis (data not shown), 352 clones (25% of the total) with differential expression were identified, from which 297 were up-regulated in I over W, whereas 55 were down-regulated. These preliminary results demonstrated that RindPdigS is enriched in genes with higher expression in infected than in wounded tissue, and confirmed that the generated cDNA macroarray is an effective efficient tool for analyzing the transcriptional responses of citrus fruit to *P. digitatum *infection.

### Identification of gene expression changes in the response of citrus fruit to ethylene, wounding and/or infection

Additional RNA samples were obtained from 'Navelina' oranges treated with either ethylene (10 ppm) (sample E) or with air (sample A) for 16 h in the absence of infection, and were processed to conduct macroarray hybridizations. The expression results obtained after image quantification and data processing were analyzed for significant changes between two independent conditions (see Materials and Methods for details). Six different comparisons were carried out, referred to as E/A, W/A, I/A, W/E, I/E and I/W. The complete set of data as log_2 _ratios for the 1536 spots can be found as Additional File 1.

**Figure 1:**
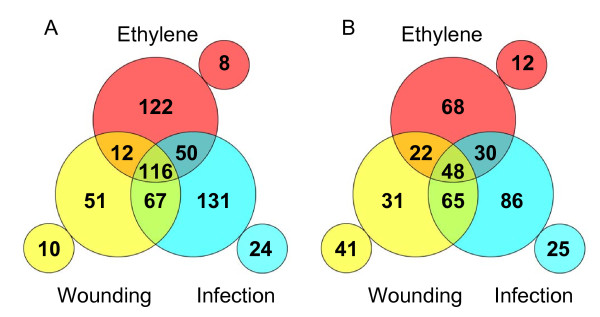
**Distribution of up and downregulated genes in 'Navelina' oranges in response to either wounding, ethylene or *P. digitatum *infection with respect to non-treated fruits**. (A) Induced clones. (B) Repressed clones. Numbers in overlapping regions indicate shared clones, numbers in non-overlapping areas denote stimulus-specific responses and numbers in small circles indicate clones specifically present (A) or absent (B) in each condition.

Only 460 ESTs did not show any significant change in any of the six comparisons, although out of this group 70 clones did not show hybridization signal in any tested condition. Fifty five ESTs were specifically present in only one treatment, whereas 123 were specifically absent in one condition. The remaining clones that showed differential expression in at least one condition were grouped into distinct gene expression classes. A representative illustration of the distribution of induced or repressed clones in the three experimental conditions respect to the Air control is shown in Fig. 1. For instance, 388 (27%) clones were found to be induced in response to *P. digitatum *infection over the control, from which 166 also showed induction by ethylene, whereas 131 showed specific induction in response to pathogen challenge and 24 were only detected in infected peel tissue. The array contained 254 down-regulated ESTs during infection as compared to the air control. Expression classes additional to those summarized in Fig. 1 were also identified since some ESTs were up-regulated in some of the conditions and down-regulated in others (see Additional File 1). Noteworthy, 25 of the clones repressed by infection were induced in response to ethylene treatment. On the other hand, among clones induced during infection there were 22 whose expression was repressed by ethylene and 7 by wounding. Thus, although the general pattern corresponded to transcripts induced by infection, ethylene or both stimuli, there was a minor but significant number of clones that deviate from such general response, revealing the complexity of gene regulation in citrus fruit in response to pathogen infection.

### Sequence analysis of RindPdigS

A total of 388 sequences were obtained from 371 RindPdigS clones. These clones included representatives from each one of the different expression patterns, as well as all the clones that showed a high hybridization signal and those that showed an infection/wounding ratio higher than 3. Only 15 of the sequenced clones contained two inserts and 1 clone contained three inserts (a low proportion of 4.3% of concatemer clones). A total of 198 sequences were assembled into 62 contiguous sequences (contigs from P-01 to P-62, which are clusters containing more than one EST), while 167 sequences were singletons (solitary or non-clustered ESTs), accounting for a total of 229 unigenes (Table 1). Using the BLASTX algorithm, DNA sequences were searched against the NCBI non-redundant protein database. The complete set of sequence assignments can be found as Additional File 2. We are aware that some of the identified unigenes could be derived from the same mRNA. Therefore, we conducted sequence analyses with homologous gene sequences in databases to search for such cases among the most abundant or representative contigs. Hence, contigs P-07 and P-08 align perfectly to the *C. sinenis CsACO *gene (Acc. No. AF321533) and are flanked by *Rsa*I restriction sites, the enzyme used to digest the cDNA during construction of the subtracted cDNA library. A similar situation occurred with contigs P-03, P-27 and clone N14G04. These three unigenes align contiguously to the CFGP unigen aCL246Contig2, which shows high similarity to an oxidoreductase that contains a FAD-binding domain.

**Table 1:** Summary of the subtracted cDNA library RindPdigS from orange and the non-subtracted cDNA library RindPdig24 from mandarin

	RindPdigS	RindPdig24
Total sequences	388	1152
Valid sequences	365	1116
Sequences in clusters	198	352
Total number. of clusters	62	143
Singleton sequences	167	764
Total number of unigenes	229	907
Redundancy (%)	37.3	18.7

As a first step in characterizing the transcriptional profile of infected fruit we identified the contigs containing the highest number of ESTs. Table 2 shows the 16 most abundant contigs, and presents the gene expression profile of each gene under the different examined conditions. In general, profiles showed an induction of expression with infection and to a lower, but significant level, also with wounding and ethylene treatment. As a remark of this general response we have summarized the data on the sequenced EST with the highest ratio of expression in infected versus wounded samples (Table 3). Tables 2 and 3 are two complementary ways of representing the transcriptional response of citrus fruit to *P. digitatum *infection. Annotation of unigenes shown in Tables 2 and 3 indicate that many of these ESTs show homology to plant genes involved in secondary metabolism, such as those corresponding to 3-deoxy-D-arabino-heptulosonate 7-phosphate (DAHP) synthase (contig P-01), FAD-binding proteins, caffeine synthase, tropinone reductase or to different cytochrome P450.

**Table 2:** Major properties of the most abundant ESTs in RindPdigS

Cluster	Best BlastX match	Accession No.	E-value	No ESTs (RindPdigS)	No ESTs (RindPdig24)	Log_2_(E/A)	Log_2_(W/A)	Log_2_(I/A)	Transcriptional profile^a^
P-02	No blast match	-	-	11	0	-0.07 ± 0.28	-0.26 ± 0.53	1.51 ± 1.41	I > EWA
P-03	FAD-binding domain containing protein [*Arabidopsis thaliana*]	NP_181025.1	1.E-08	11	0	0.52 ± 0.27	0.59 ± 0.60	3.11 ± 0.42	I>EW > A
P-01	DAHP synthase [*Vitis vinifera*]	AAN77866.1	3E-26	10	2	0.99 ± 0.24	1.65 ± 0.34	2.62 ± 0.32	I>W>E > A
P-07	ACC oxidase [*Citrus sinensis*]	AAG49361.1	5E-36	9	4	*	*	*	I>EW > A
P-10	Embryo-abundant protein-related [*Arabidopsis thaliana*]	NP_181669.1	3E-25	7	0	1.85 ± 0.26	**	2.41 ± 0.40	I>E > WA
P-09	Ribosomal L18ae protein family [*Aspergillus nidulans*]	EAA66532.1	9E-32	6	0	-0.15 ± 0.13	**	0.93 ± 0.80	I > EWA
P-04	Cystinosin homolog [*Arabidopsis thaliana*]	P57758	5E-35	5	0	1.46 ± 0.13	-0.17 ± 0.22	-0.05 ± 0.28	E > IWA
P-05	No blast match	-	-	5	0	0.17 ± 0.24	-0.01 ± 0.25	0.49 ± 0.79	I > EWA
P-11	homogentisic acid geranylgeranyl transferase [*Triticum aestivum*]	AAP43912.1	8E-12	5	3	0.85 ± 0.80	0.82 ± 0.46	1.90 ± 0.31	I>EW > A
P-06	ABC transporter family protein [*Arabidopsis thaliana*]	NP_181179.2	1E-19	4	0	-0.36 ± 0.17	0.01 ± 0.06	1.28 ± 0.20	I > EWA
P-08	ACC oxidase [*Citrus sinensis*]	AAG49361.1	2E-60	4	4	*	*	*	I>EW > A
P-14	Cyclase family protein [*Arabidopsis thaliana*]	NP_567957.1	2E-37	4	0	0.80 ± 0.31	-0.38 ± 0.35	-0.67 ± 0.24	E > IWA
P-15	40 S ribosomal protein S5 [*Aspergillus nidulans*]	XP_658447.1	3E-30	4	2	*	*	*	I > EWA
P-46	Dicyanin [*Lycopersicon esculentum*]	AAF66242.1	3E-33	4	0	0.05 ± 0.01	1.77 ± 0.59	2.66 ± 0.37	I>W > EA
P-53	No blast match	-	-	4	4	-0.05 ± 0.12	-1.38 ± 0.19	-1.41 ± 0.10	EA > IW
P-62	Caffeine synthase *[Theobroma cacao*]	BAE79730.1	2E-20	4	0	*	*	*	I

^a^Relative expression level after treatment with air (A), ethylene (E), wounding (W) or *P. digitatum *infection (I) as determined by macroarray hybridazation.

*No expression was detected in control tissue.

**No expression was detected in wounded tissue.

**Table 3:** RindPdigS clones showing the highest induction in response to *P. digitatum *infection according to macroarray hybridization

Clone/Cluster	Best BlastX match	Accession No.	E-value	No ESTs (RindPdigS)	No ESTs (RindPdig24)	I/W macroarray^a^	I/W Northern^b^
P-18	hypothetical protein AN7411.2 [*Aspergillus nidulans*]	EAA61782.1	2E-28	3	0		
P-32	short-chain dehydrogenase Tic32 [*Pisum sativum*]	AAS38575.1	3E-33	2	0		
P-62	Caffeine synthase [*Theobroma cacao*]	BAE79730.1	2E-20	4	0		24.25
N14G12	No blast match			1	0		
N17C02	No blast match			1	0		
N17E11	expressed protein [*Arabidopsis thaliana*]	NP_190988.1	3E-06	1	0		
P-43	Cytochrome P450 79A1 (Tyrosine N-monooxygenase	Q43135	3E-29	2	0	11.54	27.93
P-27	nectarin 5 [*Nicotiana langsdorffii *x *Nicotiana sanderae*]	AAP30841.1	7E-15	3	0	9.15 ± 0.77	
P-31	ADP/ATP carrier protein [*Penicillium chrysogenum*]	BAC82547.1	1E-16	2	0	8.67	
P-26	Unknown protein [*Arabidopsis thaliana*]	AAL32635.1	2E-24	2	0	8.23	
N14G04	nectarin 5 [*Nicotiana langsdorffii *x *Nicotiana sanderae]*	AAP30841.1	4E-46	1	0	7.69	
N06E04	polygalacturonase [*Penicillium digitatum*]	BAA77297.1	5E-22	1	1	7.52	
P-10	Embryo-abundant protein-related [*Arabidopsis thaliana*]	NP_181669.1	3E-25	7	0	6.99 ± 0.77	*
N07B09	expressed protein [*Arabidopsis thaliana*]	NP_565660.1	2E-13	1	0	6.62	
P-03	FAD-binding domain containing protein [*Arabidopsis thaliana*]	NP_181025.1	1.E-08	11	0	6.32 ± 1.35	*
N08F02	probable tropinone reductase [Arabidopsis thaliana]	C84693	2E-21	1	1	6.14	2.46
N11D06	EF1-alpha translation elongation factor [Sordaria macrospora]	CAA65435.1	3E-24	1	0	5.88	
N08C11	chloroplast ribosomal protein L1 [Pisum sativum]	CAA58020.1	2E-50	1		5.56	
N07E06	dehydration-responsive protein, [Arabidopsis thaliana]	NP_174468.1	4E-83	1	0	4.58	
P-16	pleiotropic drug resistance like protein [Nicotiana tabacum])	BAB92011.1	2E-15	3	0	5.94 ± 5.64	2.86
P-21	P0468B07.6 [Oryza sativa]	NP_915910.1	7E-06	2	1	4.20 ± 0.24	7.90
N12G08	mitochondrial phosphate translocator [Medicago truncatula]	AH59632.1	2E-57	1	0	4.13	
P-02	No blast match			11	0	4.76 ± 2.08	30.72
N13E10	cellular apoptosis susceptibility protein, putative [Arabidopsis thaliana]	NP_182175.1	2E-15	1	0	4.03	7.12
P-07	ACC oxidase [Citrus sinensis]	AAG49361.1	5E-36	9	4	4.05 ± 0.61	

^a^Gene expression ratios in infected (I) *vs *wounded (W) tissue according to macroarray hybridization.

^b^Gene expression ratios in infected (I) *vs *wounded (W) tissue according to Northern hybridization.

*Expresion not detected in wounded tissue

Three of the clusters shown in Table 2 deviated from the general behavior of higher induction with infection. Induction by ethylene treatment was higher than in any other condition in the case of a cystinosin homolog (contig P-04) and a cyclase family protein (contig P-14) (Table 2). In the case of the contig P-53, with no homology to known sequences, a repression after wounding (and also infection) was concluded from our data. Noteworthy is the specific expression in infected tissue of contig P-62, which shows homology to a caffeine synthase. Thirteen out of the 16 most abundant contigs showed homology to known sequences and only three contigs presented novel sequences with no homology to known sequences. Interestingly, one of these elements is contig P-02, which contains the highest number of ESTs and shows up-regulation only in response to pathogen challenge, whereas its expression level remains unchanged after ethylene treatment or wounding. A similar expression pattern is shown by the other most abundant contig, contig P-03, which shows homology to a FAD-dependent oxidoreductase. Another highly represented gene with a high expression level in infected peel tissue shows homology to DAHP. This gene, however, is also induced by ethylene and wounding. A gene coding for an ACC oxidase, present in contigs P-07 and P-08, is the only gene previously known to be highly induced in citrus fruit challenged with *P. digitatum *[7,12]. According to the hybridization results this gene is not expressed in control fruits but it is strongly up-regulated in all other conditions, being one of the genes with highest expression level in infected fruit tissue (see Additional File 1).

Despite the low amount of fungal biomass at 24 hpi, the isolation of *P. digitatum *ESTs was expected and in fact fungal assignments were also obtained (18 clones, 6.4% from the total number of valid sequences). Although most of the fungal ESTs corresponded to ribosomal proteins, including contigs P-09 and P-15, (see Table 2), we have also isolated transcripts corresponding to a hypothetical protein (contig P-18), an ADP/ATP carrier protein (contig P-31), a polygalacturonase and a translation elongation factor, which in general show a high up-regulation in infected tissue (Table 3).

Among the sequences with the highest induction in infected tissue over wounded tissue (Table 3), we found six genes expressed only in infected peel tissue, including the fungal contig P-18. Three of them show homology to plant genes. Contigs P-62 and P-32, encoding a putative caffeine synthase and a short-chain alcohol dehydrogenase, respectively, belong to this latter category. Considering citrus genes that are expressed both in wounded and infected tissues, the highest induction level was found in contig P-43, which shows homology to a cytochrome P450. This gene showed a high up-regulation in response to pathogen infection, but its expression was not altered by either ethylene or wounding. A similar regulation pattern was found in most of the citrus genes shown in Table 3, although few genes were down-regulated by ethylene.

### Comparison of RindPdigS and RindPdig24 cDNA libraries

RindPdig24 is a non-subtracted library representing the mRNA population in the peel of mandarin fruits (*C. clementina*) 24 hpi with *P. digitatum*, and its analysis within the context of the CFGP has already been reported [20]. However, it was of interest to conduct a comparative analysis of this non-subtracted library with the subtracted library RindPdigS (Table 1). RindPdig24 contains a total of 1116 high quality ESTs, grouped into 143 contigs and 764 singletons. Remarkably, only 6 from the 16 most abundant contigs in RindPdigS are also found in RindPdig24 (Table 2). Moreover, only 41 unigenes are common to both libraries and 188 unigenes from RindPdigS (82% of the total) are not present in RindPdig24. Hence, these data confirm that RindPdigS, although more redundant than RindPdig24 (Table 1), contains distinct sequence information, likely more specific of the infection process.

**Figure 2:**
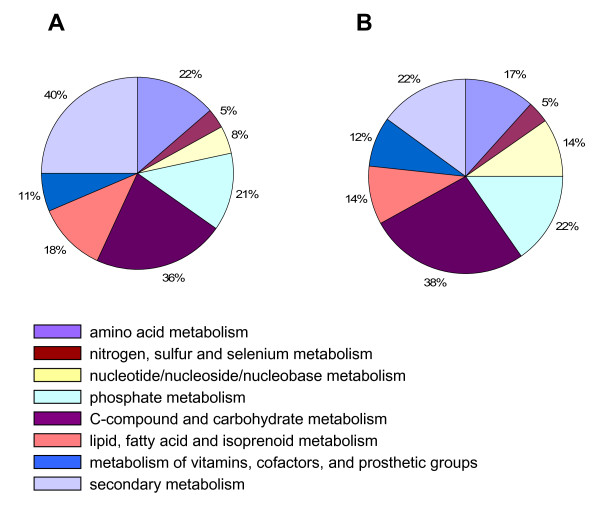
**Distribution of ESTs within the MIPS "metabolism" category in (A) the subtracted cDNA library RindPdigS from orange and (B) the non-subtracted cDNA library RindPdig24 from mandarin**.

Comparison of the distribution of ESTs into MIPS functional categories indicated differences between both libraries (Table 4). Higher abundance in RindPdigS was found in the categories "metabolism", "cellular transport", "interaction with the environment" (in this case mostly due to ethylene biosynthesis related genes) and "systemic interaction with the environment". Categories with lower abundance in RindPdigS were "cell cycle", "transcription", "protein synthesis", and "regulation of metabolism and protein function". The most relevant annotation in terms of abundance as well as differences between the subtracted and non-subtracted libraries was metabolism (Table 4 and Fig. 2). The distribution of ESTs in this category reveals that RindPdigS is enriched over RindPdig24 in genes related to secondary metabolism (40.4% *vs *21.9%), amino acid metabolism (21.9% *vs *17.0%) and lipid, fatty acid and isoprenoid metabolism (18.4% *vs *14.2%), whereas nucleotide metabolism (7.9% *vs *13.8%) is under-represented. (Fig. 2) Within secondary metabolism, that of methionine, and dehydroquinic, shikimic and chorismic acids, account for 60% of the annotations in the subtracted cDNA library.

**Table 4:** Distribution of annotated ESTs among MIPS functional categories.

Functional category	RindPdigS	RindPdig24	*A. thaliana*
Metabolism	37.6	27.7	17.3
Energy	3.6	5.5	1.6
Storage protein	0.0	0.1	0.2
Cell cycle and DNA processing	2.4	4.2	5.3
Transcription	1.8	6.0	9.4
Protein synthesis	4.2	9.6	4.7
Protein fate (folding, modification, destination)	12.7	14.7	10.9
Protein with binding function or cofactor requirement	26.7	32.3	24.7
Regulation of metabolism and protein function	0.6	2.9	2.1
Cellular transport, transport facilitation and transport routes	20.0	13.7	8.5
Cellular communication/Signal transduction mechanism	2.4	3.5	4.5
Cell rescue, defense and virulence	12.7	10.5	5.0
Interaction with the environment	14.5	10.5	5.8
Systemic interaction with the environment	6.1	3.3	2.7
Transposable elements, viral and plasmid proteins	0.0	0.1	0.3
Cell fate	1.8	1.9	1.6
Development (systemic)	3.0	3.8	3.7
Biogenesis of cellular components	2.4	4.5	5.5
Cell type differentiation	1.2	0.9	0.8
Tissue differentiation	0.6	0.9	0.3
Organ differentiation	1.2	1.0	0.9
Subcellular localization	46.7	50.5	46.6
Cell type localization	0.6	0.1	0.5
Tissue localization	0.6	0.3	0.1
Organ localization	1.2	0.1	0.1
Unclassified proteins	18.8	16.0	28.1

### Northern blot analyses of gene expression changes

**Figure 3:**
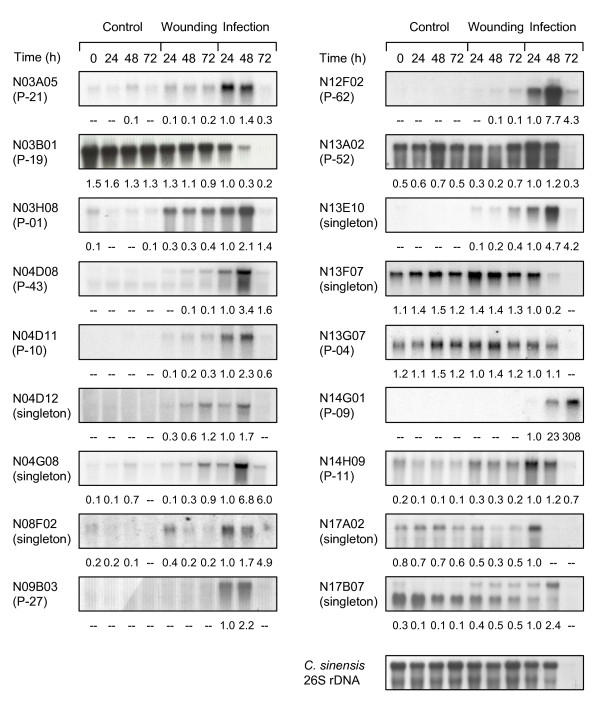
**Northern blot analysis of genes isolated from the RindPdigS library in control, wounded or *P. digitatum *-infected 'Navelina' oranges**. RNA samples were obtained from either control, wounded or *P. digitatum*-infected 'Navelina' oranges at different times after inoculation. In brackets it is indicated whether the clone is a singleton or the cluster it belongs to. Membranes were hybridized with probes corresponding to: P0468B07.6 [*O. sativa*] (N03A05), beta-carotene hydroxylase (N03B01), 3-deoxy-D-arabino-heptulosonate 7-phosphate synthase (N03H08), Cytochrome P450 79A1 (N04D08), embryo-abundant protein (N04D11), nectarin 5 (N04D12), Caffeic acid 3-O-methyltransferase 1 (N04G08), tropinone reductase (N08F02), nectarin 5 (N09B03), caffeine synthase (N12F02), GcpE (N13A02), cellular apoptosis susceptibility protein (N13E10), aquaporin (N13F07), cystinosin (N13G07), hypothetical protein AN0433.2 (N14G01), homogentisic acid geranylgeranyl transferase (N14H09), wax biosynthesis protein (N17A02) and acetyltranferase-like protein (N17B07). Hybridization with the *C. sinensis *26 S rDNA is shown in the bottom panel. Normalization of hybridization signals was carried out with respect to the hybridization signal of the *C. sinensis *rDNA. Values below the panels show the relative quantification of the corresponding hybridization signal referred to the value of the infected sample at 24 hpi. Those hybridization signals lower than two folds the intensity of background were not assessed (-).

To confirm the expression profiles obtained from macroarray hybridizations, Northern blot analysis was carried out for 38 genes selected on the basis of their expression level, expression pattern or biological significance. In this analysis we included additional time points after treatments and confirmed the differential expression in response to both wounding and *P. digitatum *infection for 31 of them (82%) (Fig. 3 and Additional File 3).

Examples of citrus genes up-regulated upon *P. digitatum *infection, and with lower or no expression in control or wounded samples, are homologs of Tyrosine N-monooxygenase (contig P-43), nectarin 5 (N04D12 and contig P-27), caffeic acid Ο-methyl transferase (N04G08), tropinone reductase (N08F02), caffeine synthase (contig P-62) and homogentisic acid prenyltransferase (contig P-11) (Fig. 3). All these annotations are related to specific pathways of amino acids and secondary metabolism.

The most common expression pattern was induction in response to pathogen infection and to a lower extent with wounding, similarly to that found in macroarray hybridization data. There are examples of highly abundant mRNAs in some (or all) of the control conditions that increase their expression upon infection, as DAHP synthase (contig P-01) or a GcpE homolog (contig P-52, involved in isoprenoid biosynthesis) (Fig. 3). Most of the citrus mRNAs have maximum accumulation at 48 hpi and decrease at 72 hpi, likely as consequence of cellular lysis and degradation of plant cell constituents because of the maceration and colonization by *P. digitatum *[12]. Nevertheless, Northern hybridization allowed the identification of additional time-course patterns of expression and also of early responsive genes upon infection, such as N17A02, involved in wax biosynthesis, or wounding, as N08F02, encoding a tropinone reductase homolog, whose expression is maximal after 24 hours of treatment (Fig. 3). Contig P-09 is an example of a fungal gene (Table 2) whose expression is detected only in infected tissue and shows a maximum expression at 72 hours after inoculation, coincident with the maximum development of the fungus throughout the rind (Fig. 3).

**Figure 4:**
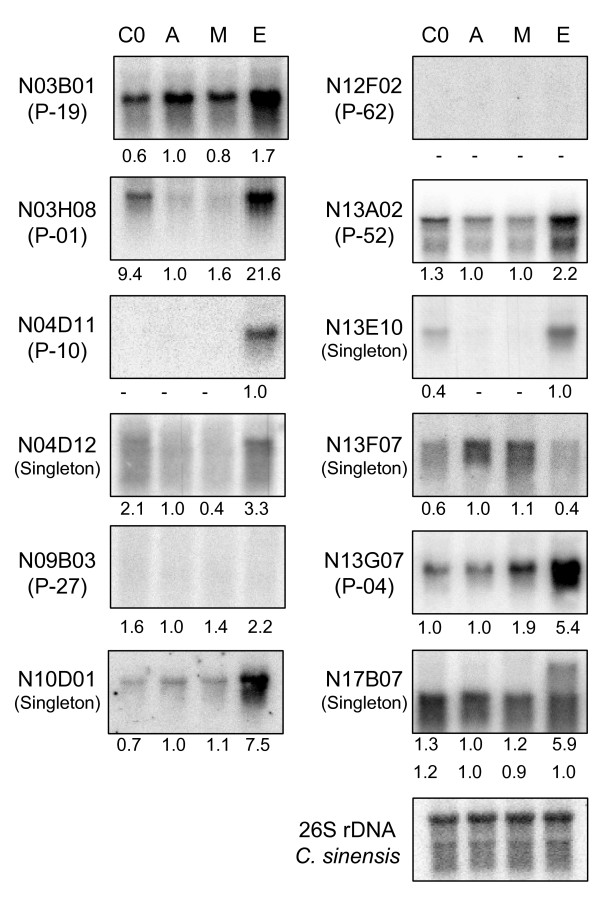
**Northern blot analysis of clones belonging to RindPdigS in *C. sinensis *Navelate fruit's peel after ethylene or 1-MCP pre-treatment**. Tissue samples were analysed before any treatment (C0) or after 16 h of treatment with air (A), 500 ppb of 1-MCP (M), or 10 ppm of ethylene (E). In brackets it is indicated whether the clone is a singleton or the cluster it belongs to. Membranes were hybridized with probes corresponding to beta-carotene hydroxylase (N03B01), 3-deoxy-D-arabino-heptulosonate 7-phosphate synthase (N03H08), embryo-abundant protein (N04D11), nectarin 5 (N04D12), nectarin 5 (N09B03), 3-phosphoshikimate 1-carboxyvinyltransferase (N10D01), Caffeine synthase (N12F02), GcpE (N13A02), cellular apoptosis susceptibility protein (N13E10), aquaporin (N13F07), cystinosin (N13G07) and acetyltranferase-like protein (N17B07). Hybridization with the *C. sinensis *26 S rDNA is shown in the bottom panel. Normalization of hybridization signals was carried out with respect to the hybridization signal of the *C. sinensis *rDNA. Values below the panels show the relative quantification of the corresponding hybridization signal referred to the value of the sample E. Those hybridization signals lower than two fold the background intensity were not assessed (-).

Northern blot analyses were also conducted to confirm expression changes induced by ethylene (Fig. 4 and Additional File 4). In general, these results confirm those obtained by macroarray hybridization. Most of the analyzed genes increased or switched on their expression after exogenous ethylene treatment, in correlation with their induction upon infection. However, examples from all the potential combinations of induction/repression after infection/ethylene were found. Thus, there are examples of ethylene-activated genes that were not up-regulated upon infection, such as contigs P-04, a cystinosin homolog, or P-19, a β-carotene hydroxylase involved in carotenoid biosynthesis (compare Figs. 3 and 4). Moreover, the β-carotene hydroxylase transcript (contig P-19) disappeared at a higher rate than what it was expected from tissue maceration.

On the contrary, there are examples of genes induced during infection that are not responsive to ethylene, as the homologs of nectarin 5 (contig P-27) and caffeine synthase (contig P-62). Interestingly, another unigene annotated as nectarin 5 homolog (singleton N04D12) was induced by ethylene and had an expression pattern distinct from P-27 (Figs. 3 and 4). Another result that points to a differential expression pattern of distinct genes from the same family is that found with probe N17B07, which codes for an acetyl transferase homolog and that unexpectedly shows two mRNA hybridization bands: the upper is turned on by ethylene, wounding and to a higher extent by infection, while the lower is insensitive to ethylene and in fact decays after wounding and infection (Figs. 3 and 4). Selected examples were found of genes repressed by ethylene, being the most representative the one coding for a homolog of an aquaporin involved in drought stress (singleton N13F07).

## Discussion

As an approach to understand the response of citrus fruit to *P. digitatum *infection we have used the SSH procedure [21] to obtain a cDNA library, RindPdigS, enriched in genes with increased expression in infected tissue. Comparison of sequences from RindPdigS with those of the non-subtracted cDNA library RindPdig24 (Table 3) indicated that RindPdigS contains distinct sequence information and reinforces the convenience of using subtractive libraries to gain additional knowledge of specific biological processes. A factor that might explain the limited overlap between these two libraries could be the normalization step included in the SSH procedure, which enables the enrichment in genes with low but differential expression. Although these two cDNA libraries were obtained from two different *Citrus *species, *C. sinensis *and *C. clementina *for RindPdigS and RindPdig24, respectively, previous work has shown a high level of identity between homologous genes from these closely related *Citrus *species [20], as we have also found by comparing common unigenes between both libraries, whose identity was usually higher than 99%.

RindPdigS is now included within the Spanish CFGP http://bioinfo.ibmcp.upv.es/genomics/cfgpDB/. In fact, our data indicate that 27% of the unigenes of RindPdigS are not found in any other of the CFGP libraries, which contain 27551 unigenes, including the closely related RindPdig24 library. We recognize that this could be an overestimate, but it should be considered that although the CFGP is focused on *C. clementina *as plant material, it also contains libraries from *C. sinensis*, and that contig analysis parameters within CFGP are expected to cluster together sequences from the different *Citrus *species covered by the project. Moreover, 21% of the sequences of RindPdigS do not show BlastX match with the NCBI non redundant database, and 4% of the sequences match proteins of unknown function. Therefore RindPdigS is a rich source for the identification of new genes likely involved in the response of citrus fruit to pathogen attack. In fact, this tool has allowed the design and use of a cDNA macroarray that enabled to gain novel conclusions. Most importantly, our data indicate that citrus fruits react to pathogen infection in a regulated and coordinated manner, by upregulating genes involved in the synthesis of specific classes of compounds with potential antimicrobial activity.

### Regulation of gene expression changes upon P. digitatum infection

Ethylene plays a pivotal role in many plant developmental and stress processes. In the case of defense against pathogen infection, the role of ethylene is complex and somewhat controversial [22,23]. Diseased citrus fruits produce significant amounts of ethylene, both from fungal and plant origin [11,12]. We have previously shown that treatment of citrus fruits with either ethylene or the ethylene perception inhibitor 1-MCP prior to infection modifies the outcome of the interaction, suggesting a contribution of ethylene to defense against infection [12].

Ethylene-induced genes are highly present among those induced by wounding and infection. Also, from the total ESTs that change their expression in response to pathogen infection (either induced or repressed), approximately 40% of them are affected by application of ethylene in absence of infection (Fig. 1). Conversely, approximately 50% of the ESTs responsive to exogenous ethylene are affected by infection. Therefore, overall results indicate that although ethylene plays an important role in the regulation of the response of citrus fruits to *P. digitatum *infection, is only a part of a complex network and other factors/signals are also likely involved.

Noteworthy examples of opposite regulation by ethylene and infection are observed in an EST from the carotenoid biosynthetic pathway, which is highly induced by ethylene (contig P-19) [24] but whose mRNA decays quickly after infection despite the high ethylene emission, or those of specific alkaloid ESTs (contigs P-27 and P-62), which on the contrary are rather specific of infection but are not responsive to ethylene (see Figs. 3 and 4). A remarkable example is represented by two distinct unigenes putatively related to the benzylisoquinoline alkaloids biosynthetic pathway (see below): P-03 (and also P-27, which likely derives from the same cDNA sequence) are specifically expressed upon fungal infection but do not react to the hormone, while N04D12, which codes for a very similar protein, is induced by ethylene, wounding and fungal infection. This example illustrates the existence of a regulation that redirects secondary metabolism to the biosynthesis of specific compounds depending on the stress and the signals involved.

Interestingly, ESTs potentially involved in signal(s) perception and transduction were also identified among RindPdigS sequences. Significant examples are three independent GTP-binding proteins (including contig P-49), four protein kinases (including contigs P-24 and P-29), two calcineurin-like phosphoesterases, one 14-3-3 like-protein, and three independent transcription factors. These data would suggest that the fruit reacts in a coordinated manner by inducing signaling cascades involved in the regulation of specific genes as a response to *P. digitatum *attack. However, only the complexity of this response is envisioned, and the molecular mechanisms underlying this response are far from being understood.

### Involvement of secondary and amino acid metabolisms in the response of citrus fruit to fungal infection

**Figure 5:**
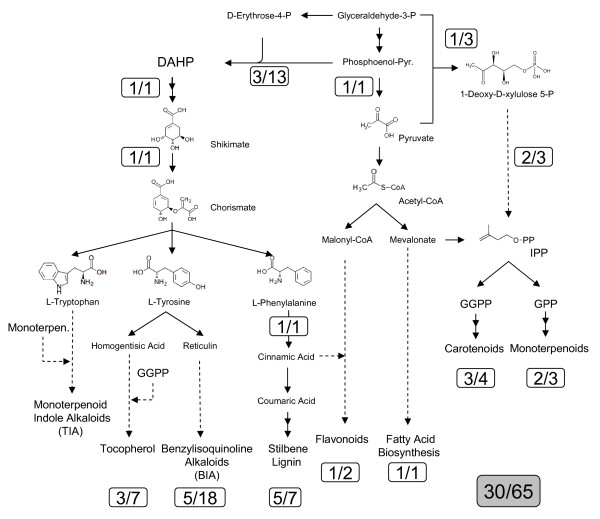
**Distribution of RindPdigS clones/unigenes within secondary metabolism pathways**. Schematic diagram showing the distribution of RindPdigS clones/unigenes within secondary metabolism pathways and the connections among them. The first number within a box indicates the number of unigenes in each part of the route and the second number reflects the clones belonging to these unigenes. The total number of clones/unigenes is shown at the bottom in a grey box.

The annotation of RindPdigS clones revealed that secondary and amino acid metabolisms are involved in the response of citrus fruits to *P. digitatum *infection (Tables 2, 3, and 4). In fact, 30 of the unigenes (13.1% of 229) and 65 of the total number of clones (17.8%) of RindPdigS could be allocated in a schematic diagram that covers connections among some of these metabolic pathways (Fig. 5). Most of these ESTs have confirmed induction of expression by Northern blot (Fig. 3 and Additional File 3) and/or macroarray hybridizations (see Additional File 1).

The shikimate biosynthetic pathway [25] is over-represented in RindPdigS. Three different unigenes (including the abundant contig P-01) are *Rsa*I fragments from a DAHP synthase homolog, and account for 13 clones. It is well known that plant DAPH synthase is induced by wounding and pathogen attack [26]. There are two additional RindPdigS ESTs involved in the synthesis of shikimate and chorismate, respectively. The latter is the precursor of the aromatic amino acids tryptophan, tyrosine and phenylalanine (Fig. 5). Plants use these amino acids as precursors of a large number of secondary metabolites that include defense compounds [27]. Indeed, RindPdigS contains sequences annotated as O-methyltransferases, N-methyltransferases, cytochrome P450 monooxygenases and hydroxylases, FAD oxidoreductases or decarboxylases that could be involved in metabolic conversions that stem from the aromatic amino acids to produce alkaloids, phenylpropanoids, and tocopherols (see below for examples).

Tyrosine can go through a sequence of enzymatic modifications to produce dopamine and 4-hydroxyphenylacetaldehyde, which are condensed to originate the precursor of benzylisoquinoline alkaloids (BIAs), including reticuline [28]. One step in the synthesis of the BIA berberine is catalyzed by a FAD-dependent oxidoreductase, named berberine bridge enzyme (BBE) as it converts the N-methyl group of (S)-reticuline into the methylene bridge moiety of (S)-scoulerine, a conversion that is unique in nature [29]. BBE is a key branchpoint enzyme in the biosynthesis of certain BIAs [29]. Four distinct unigenes that arise from 16 clones were annotated as BBE-like sequences. One of them is the most abundant contig in RindPdigS (Table 2, contig P-03), and was up-regulated during infection as it could be confirmed with Northern blot and macroarray data. The significance of this class of alkaloids in the defense response against pest and pathogen attack is well known [30-32].

Although BBE catalyses a specific reaction in the biosynthesis of BIAs, other members of this family would be expected to act on a range of substrates, as recently demonstrated by the functional characterization of the tobacco nectarin 5 protein, a BBE homolog that has carbohydrate oxidase activity [33]. Thus, the actual enzyme substrate and reaction for these citrus proteins remain unclear, as no BIA has ever been shown to be produced in citrus. It has to be noted that genes similar to those participating in the biosynthesis of BIAs have also been isolated in other plants that do not produce these alkaloids [29]. Interestingly, several citrus nectarin 5 homolog genes are also upregulated in *C. sinensis *plants infected with the bacterial pathogens *Xanthomonas axonopodis *and *Candidatus *Liberibacter asiaticus, responsible of citrus canker and Huanglonbing, respectively, as determined by microarray hybridization [34,35].

Isoprenoids are a large group of secondary metabolites, and their biosynthesis depends on the presence of the key precursor isopentenylpyrophosphate (IPP). In plants, two alternative pathways are involved in IPP biosynthesis, the cytosolic mevalonic acid (MVA) route and the plastidic methylerythritol phosphate (MEP) one, whose first committed step is the synthesis of deoxyxylulose-5-phosphate (DXP) [36]. RindPdigS library contains genes belonging to the MEP pathway, as contig P-17, which codes for the enzyme synthesizing DXP, and two unigenes annotated as GcpE-like sequences. Our results showed that both DXS and GcpE are highly expressed genes in the fruit's peel under all tested conditions, and that reach higher mRNA accumulation upon fungal infection. Two additional sequences found in RindPdigS seem to be involved in the production of monoterpenoids from geranyl diphosphate (GPP): one with homology to 10-hydroxygeraniol oxidoreductase (singleton N06D12) and another with similarity to geraniol 10-hydroxylase (G10 H, contig P-48). According to the macroarray data, only the first one showed an induction upon infection. Interestingly, G10 H has been involved in (mono)terpenoid indole alkaloid (TIA) biosynthesis [37], which proceeds through condensation of monoterpenes and tryptamine, a derivative of tryptophan (Fig. 5) [28]. It has also been shown that coordinated expression of MEP pathway genes and G10 H would be required for the effective biosynthesis of TIAs [38]. Moreover, fungal elicitors are strong inducers of TIA production in *Cataranthus roseus *[39].

Additional isoprenoids are carotenoids and tocopherols (Fig. 5), whose biosynthesis is derived from IPP through geranylgeranylpyrophosphate (GGPP) [40]. Carotenoid biosynthesis is highly upregulated in colored citrus fruits [41] but seems to be repressed shortly after inoculation with *P. digitatum*, as indicated from results of the β-carotene hydroxylase contig P-19 derived from RindPdigS (Fig. 3, N03B01) and of phytoene synthase (data not shown). In the case of tocopherol biosynthesis, the GGPP moiety is reduced and transferred to homogentisate (HGA), a distinct derivative of tyrosine, to produce the precursor for tocopherol cyclation. The coordinated actions of tocopherol cyclase and S-adenosylmethionine methyl transferases produce distinct tocopherol forms. Both HGA GG Transferase (contig P-11 and clone N17B10) and tocopherol cyclase (clone N14C01) are found in RindPdigS. Contig P-11 is induced upon wounding and has early upregulation during infection (Fig. 3). Considering the down regulation of carotenoid biosynthetic genes, these data suggest that IPP and GGPP flux would be re-directed upon infection towards the synthesis of tocopherols and also monoterpenoid-derived TIAs, instead of carotenoids.

ESTs with homology to genes involved in the biosynthesis of two additional classes of plant alkaloids are also present in RindPdigS. The presence of a tropinone reductase EST (clone N08F02, Fig. 3) suggests the involvement of tropane alkaloids in the response of citrus fruit to *P. digitatum *infection. Although there are no evidences of the association of these alkaloids with disease resistance, tropane alkaloids production is induced upon elicitation with fungal cell walls, oligogalacturonides and also in response to exogenous MeJA and SA [42,43]. Moreover, the same tropinone reductase homolog gene is induced in citrus plants challenged with *X. axonopodis *[35]. This EST showed a clear early induction in response to wounding and a greater induction upon infection. The involvement of purine alkaloids is suggested by the presence of a caffeine synthase EST (contig P-62), from the biosynthetic pathway of caffeine from xanthosine. This sequence showed a remarkable infection-specific response (Fig. 3). As a matter of fact, the four ESTs that form this contig were selected for sequencing from the group of ESTs that were expressed only in infected tissue, the lack of signals in the controls precluded statistical analysis of expression in the macroarray data (Table 2).

Phenylpropanoids is the other major group of secondary metabolites that is overrepresented in the RindPdigS library. It is well known that phenylpropanoid production suffers rapid and drastic changes in response to pathogen infection [44]. Phenylalanine-ammonia lyase (PAL) is the key entry point enzyme into the biosynthesis of phenylpropanoids from phenylalanine and is known to be induced upon stress conditions [45]. *P. digitatum *enhanced *PAL *mRNA accumulation in citrus fruits [1,7,12], and in fact this was one of the 16-times replicated controls included in the array. As expected, RindPdigS also contains an EST corresponding to the citrus *PAL *gene, as well as two distinct enzymes involved in the conversion of 4-coumarate to sinapate in the initial reactions of the phenylpropanoid pathway, as clone N04G08 (Caffeic acid 3-O-methyl transferase), which showed a specific expression during infection as confirmed by Northern hybridization (Fig. 3.). Additional ESTs are related to the biosynthesis and metabolism of lignin, and also to that of other phenylpropanoid-derived compounds, such as anthocyanins and flavonoids, including enzymes involved in the modification or transport of these compounds, such as glycosyltransferases (contigs P-23 and P-37), or plant membrane ABC transporters (contigs P-06 and P-16 and singletons N13G01 and N14B03), which may participate in an active process to secrete defensive metabolites [46-48].

It is recognized that the presence of secondary metabolites genes does not necessary imply the ability of a plant to produce those compounds [29]. However, it is also true that some of the alkaloids highlighted in this study have already been identified in citrus, as is the case of caffeine [49]. Further investigations are required to determine the participation and significance of alkaloids in the response to fungal attack in citrus fruit by both compatible and non-compatible pathogens. We should bare in mind that *P. digitatum *is the most successful pathogen of citrus fruit. Hence, it must be able to overcome the plethora of secondary metabolites that the fruit seems to synthesize in order to deter its progression. However, it remains to be elucidated whether these compounds play a role in non host-pathogen defense restricting the progress of pathogens that are not able to infect citrus fruit.

## Conclusions

The present study has identified a set of citrus genes that are preferentially upregulated in the rind of citrus fruit infected with *P. digitatum *with respect to mock-inoculated fruits. Macroarray hybridization experiments have shown that approximately half of the genes responsive to *P. digitatum *infection are also responsive to wounding or ethylene, although some genes related to secondary metabolism are only induced upon pathogen challenge, such as those showing similarity to caffeine synthase, tropinone reductase or berberine bridge-like. Overall results indicate that *P. digitatum*-infected citrus fruit redirect primary metabolism towards the synthesis of alkaloids, phenylpropanoids and isoprenes.

## Methods

### Biological material

Mature oranges (*Citrus sinensis *L. Osbeck) from the cultivars 'Navelina' and 'Navelate' harvested from a commercial orchard in Lliria (Valencia, Spain) were used throughout this study.

*Penicillium digitatum *Sacc. isolate PHI-26 [50] was cultured on potato dextrose agar (Difco) plates at 24°C. Conidia were collected from 1-week-old plates by scraping them with a sterile spatula, and transferring them to sterile water. Conidia were then filtered and titrated with a hemacytometer.

### Fruit inoculation

After harvesting, fruits were washed, disinfected and dried immediately as described previously [50]. 'Navelina' fruits were first wounded by making punctures with a nail (approximately 5 mm in depth) and 10 μl of a *P. digitatum *conidia suspension (10^6 ^conidia ml^-1^) were applied to each wound. Control mock-inoculations were carried out with 10 μl of sterile water. Additional controls consisted of fruits that were not wounded. After inoculation, fruits were maintained at 20°C and 90% relative humidity. At either 0, 24, 48 or 72 h post-inoculation (hpi) peel tissue discs of 5 mm in diameter around the inoculation site (containing flavedo and albedo, but not pulp) were sampled by using a cork borer. Tissue was frozen in liquid nitrogen, grounded to a fine powder and stored at -80°C for RNA extraction.

### Pretreatment of fruit with ethylene or 1-MCP

In some experiments 'Navelina' and 'Navelate' fruits were treated with 10 ppm of ethylene or 500 ppb of 1-methyl cyclopropene (1-MCP, Rohm and Haas) for 16 h at 20°C as described previously [12]. Controls consisted of fruits treated with atmospheric air.

### Generation of a subtracted cDNA library

Total RNA was extracted from fruit peel discs as described previously [1]. Poly(A)^+ ^RNA was purified from total RNA using the Dynabeads mRNA purification kit (Dynal) following the manufacturers' instructions. One microgram of poly(A)^+ ^RNA was used to synthesize double-stranded cDNA using the Smart PCR cDNA synthesis Kit (Clontech Laboratories) according to the protocol supplied by the manufacturer. Triplicate 100 μl amplification reactions were set up for each sample; 15 cycles were determined to be optimal for the amplification of the double-stranded cDNA.

Suppression subtractive hybridization (SSH) [21] was performed with the Clontech PCR-Select cDNA subtraction kit (Clontech Laboratories) using cDNAs derived from either mock-inoculated fruits (W) or fruits inoculated with *P. digitatum *(I) and subsequently held for 24 h at 20°C as 'driver' and 'tester' material, respectively. Primary and secondary PCR amplifications were performed with Advantage 2 cDNA polymerase mix (Clontech Laboratories) using 27 and 15 cycles respectively. The resulting pool of subtracted cDNA fragments was cloned into the *EcoR*V site of plasmid BlueScript II SK+ and introduced into *Escherichia coli *DH5α through electroporation. Colonies were selected on LB agar plates supplemented with ampicillin and X-GAL to allow for a white-blue screening.

### Description of the cDNA macroarray

A total of 1436 colonies from the subtracted cDNA library were randomly picked and the corresponding plasmid inserts were amplified by colony PCR using M13 forward and reverse primers. Reactions consisted of a first denaturation step at 94°C for 5 min followed by 30 cycles at 94°C for 30 sec, 56 C for 45 sec and 72°C for 90 sec. A final extension step was conducted for 10 min at 72°C. To complete the 1536 spots available in the array (12 × 8 × 16), 100 positive and negative controls were incorporated. These included genes known to be involved in fruit responses to stress (e.g. ACC synthase, ACC oxidase and PAL) [12], as well as negative controls with either no insert, unrelated cDNA inserts or PCR reactions conducted with water as template. Samples were provided in sixteen 96-well plates to a macroarray spotting external service (Universitat de València, http://scsie.uv.es/chipsdna) and replicate nylon membranes were prepared.

### Macroarray hybridization and data analysis

[α^33^P] dCTP-labelled single-strand cDNAs were synthesized from 20 μg of total RNA by reverse transcription using anchored oligo(dT)_20_VN primer and Superscript III Reverse Transcriptase (Invitrogen) following standard protocols. The incubation was allowed to proceed for 120 minutes at 50°C. RNA was degraded by alkaline lysis, and subsequently samples were neutralized. Unincorporated [α-^33^P] dCTP was removed with MicroSpin S-300HR columns (GE Healthcare). ^33^P incorporation was quantified via liquid scintillation. The final concentration of labelled cDNA was adjusted to 3 × 10^6 ^dpm/ml with TE.

Filter hybridization and stripping were carried out as previously described [51]. Filters were pre-hybridized for at least 1 h at 65°C with 5mL of SSC 5X, Denhart's 5X, SDS 0.5%, and herring sperm DNA 100 μg/ml. Labeled cDNA was then added and hybridization was allowed to proceed at 65°C for 16 hours. Membranes were washed once for 20 min at 65°C with 2X SSC, 0.1% SDS and then twice with 0.2X SSC, 0.1% SDS for 30 min at the same temperature. Filters were wrapped in a plastic film and exposed to an imaging plate (BAS MP 2040, Fuji Film). Images were acquired with a high-resolution FLA-3000 scanner (Fuji Film). Spot intensities were quantified with the program Array Vision 7.0 (Imaging Research Inc.). For each spot, signal intensity was determined as the background-corrected ARM density value. Signal values lower than 1.5 fold the local background or lower than the average plus two standard deviation of the sixteen vector spots were not considered for further analysis. Three independent hybridizations were performed for each condition.

Data were analyzed with the ArrayStat 1.0 software (Imaging Research Inc.) assuming that conditions were independent and discarding those genes that only had one valid data out of the three replicates. Data were log_10 _transformed before normalization across replicates, within conditions. Normalization across different conditions was then performed. To identify cDNA clones that were differentially expressed among conditions a z-test for two independent conditions was performed with a nominal alpha value of 0.05 and false discovery rate correction.

### Sequencing and sequence analysis

Plasmids were isolated by the alkaline lysis method either on an individual basis using the GenElute Plasmid Miniprep (Sigma) kit or in a 96 mutilwell format using the Montage Plasmid Miniprep_96 _kit (Millipore). Single pass sequencing reactions were carried out using an ABI 3100 capillary automatic sequencer (Applied Biosystems) with fluorescent dye terminator technology. Nucleotide sequences have been deposited in the dbEST division of GenBank under accession numbers FC921755 to FC922049 and GR312940 to GR313000.

Raw sequences were obtained from chromatograms. Vector masking and trimming off primers used for cDNA construction were performed with the program Seqtools 8.4 http://www.seqtools.dk. Sequences that had less than 50 non-vector good-quality bases after trimming were discarded. Assembly of reads into contigs to estimate the redundancy of the ESTs and obtaining the unigene set was performed using the same program. Homology searches were conducted with the BlastX algorithm [52] against the National Center for Biotechnology Information (NCBI) non redundant protein database using default parameters and an arbitrary threshold of 10^-5 ^for the e-value. Functional classification for each sequence was performed with the Munich Information Center for Protein Sequences (MIPS) dataset according to the functional categorization of the corresponding *A. thaliana *ortholog http://mips.helmholtz-muenchen.de/proj/funcatDB

### Northern blot analysis of mRNA accumulation

Total RNA was electrophoresed through a formaldehyde gel and transferred to a Hybond N^+ ^membrane (GE Healthcare) following standard protocols. cDNA clones used as probes were labeled with [α-^32^P] dATP by using the Strip-EZ PCR Kit (Ambion). Prehybridization and hybridization were performed with ULTRAhyb hybridization buffer (Ambion) at 42°C following the manufacturer's instructions. Filters were washed once for 10 min with 2% SSC and 0.1% (w/v) SDS at 42°C, once for 30 min with 2X SSC, 0.1% (w/v) SDS at 55°C, and twice for 30 min with 0.1X SSC, 0.1% (w/v) SDS at 55°C. Membranes were then exposed to an imaging plate (BAS MP 2040, Fuji Film) for different periods of time. Hybridization signals were recorded with a FLA-3000 laser scanner (FujiFilm) and assessed with the software Image Gauge 4.0 (Fuji Film). Probes were stripped off using the Strip-EZ PCR Kit (Ambion) following the manufacturer's instructions.

## Authors' contributions

PST constructed the subtracted library and prepared amplicons for spotting. LGC, SA and JFM conducted fruit experiments, sequencing and macroarray and Northern hybridizations. LGC and JFM analyzed the data. LGC, SA and JFM draft the manuscript. LGC, JFM and LZ conceived and supervised the work. All authors read and approved the final manuscript.

## Supplementary Material

Additional file 1 **Macroarray hybridization results from 'Navelina' oranges treated with air (A) or with 10 ppm of ethylene (E) for 16 h, or 24 h after wounding (W) or inoculation with *P. digitatum *(I)** . Click here for file

Additional file 2 **Sequence annotation of RindPdigS clones**. Click here for file

Additional file 3 **Northern blots analysis of RindPdigS genes in control, wounded or *P. digitatum *infected 'Navelina' fruits at different time points after inoculation**. In brackets it is indicated whether the clone is a singleton or the cluster it belongs to Hybridization with the *C. sinensis *26 S rDNA is shown in the bottom panel. Normalization of hybridization signals was carried out with respect to the hybridization signal of the *C. sinensis *rDNA. Values below the panels show the relative quantification of the corresponding hybridization signal referred to the value of the infected sample at 24 hpi. Those hybridization signals lower than two folds the intensity of background were not assessed (-). Click here for file

Additional file 4 **Northern blot analysis of clones belonging to RindPdigS in *C. sinensis *Navelate fruit's peel after ethylene or 1-MCP pretreatment**. Tissue samples were analysed before any treatment (C0) or after 16 h of treatment with air (A), 500 ppb xof 1-MCP (M), or 10 ppm of ethylene (E). In brackets it is indicated whether the clone is a singleton or the cluster it belongs to. Hybridization with the *C. sinensis *26 S rDNA is shown in the bottom panel. Normalization of hybridization signals was carried out with respect to the hybridization signal of the *C. sinensis *rDNA. Values below the panels show the relative quantification of the corresponding hybridization signal referred to the value of the sample E. Those hybridization signals lower than two fold the background intensity were not assessed (-). Click here for file
