# Supplementary figures and images for: A transcriptomic approach highlights induction of secondary metabolism in citrus fruit in response to Penicillium digitatum infection
Source: BMC Plant Biol. 2010 Aug 31;10:194. doi: 10.1186/1471-2229-10-194 (PMC2956543; doi:10.1186/1471-2229-10-194)

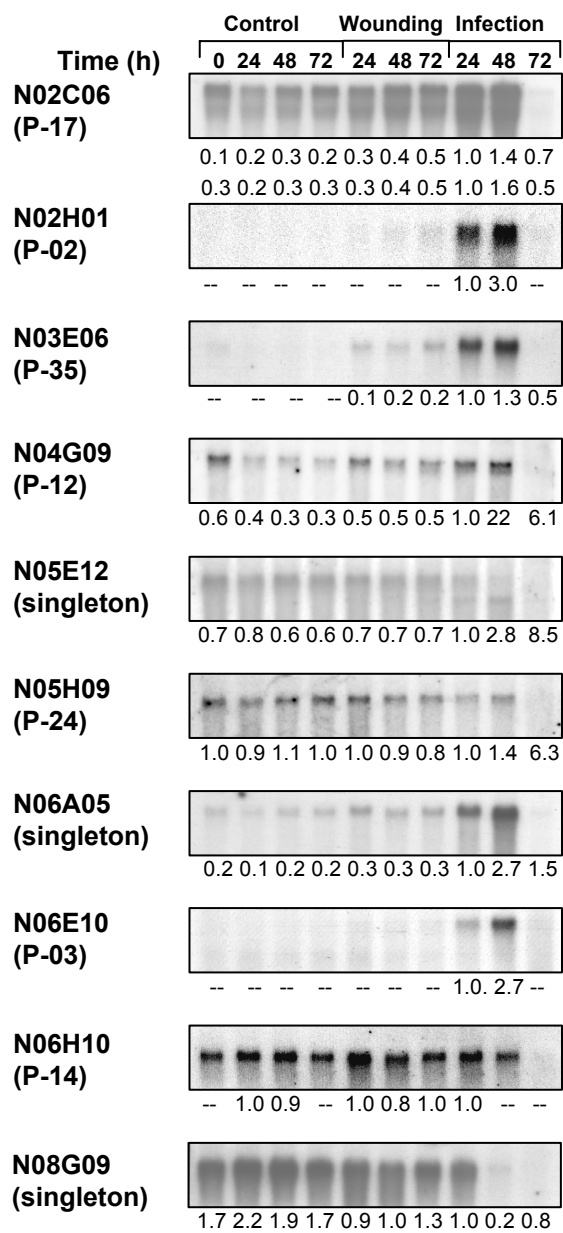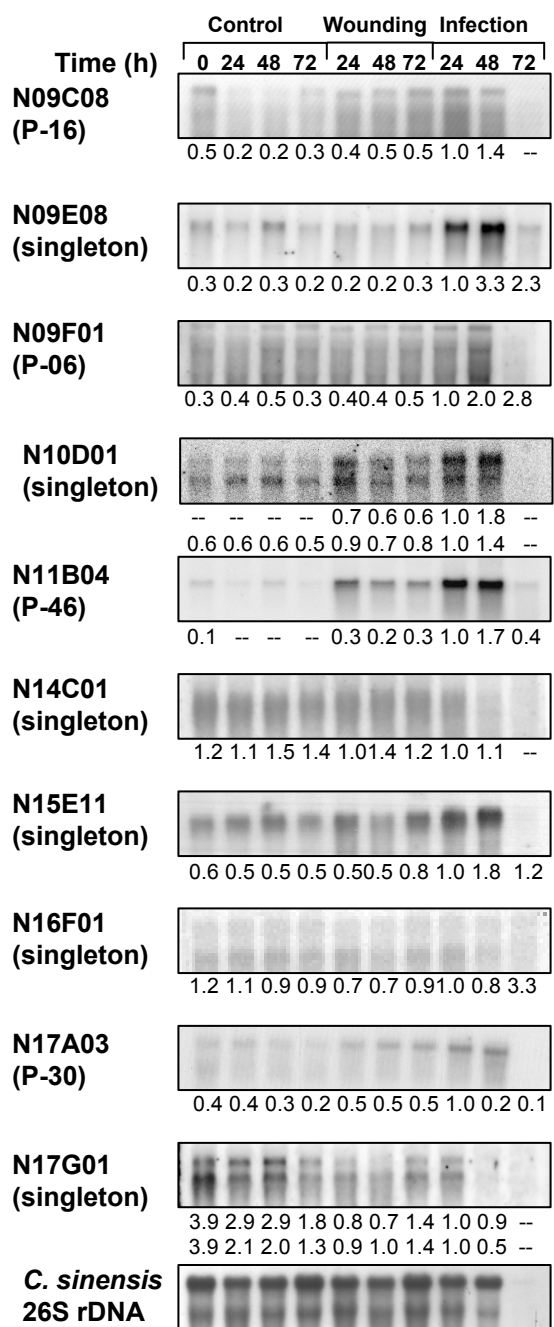

Supplement: Additional file 3 — Northern blots analysis of RindPdigS genes in control, wounded or P. digitatum infected 'Navelina' fruits at different time points after inoculation. In brackets it is indicated whether the clone is a singleton or the cluster it belongs to Hybridization with the C. sinensis 26 S rDNA is shown in the bottom panel. Normalization of hybridization signals was carried out with respect to the hybridization signal of the C. sinensis rDNA. Values below the panels show the relative quantification of the corresponding hybridization signal referred to the value of the infected sample at 24 hpi. Those hybridization signals lower than two folds the intensity of background were not assessed (-). [file 1471-2229-10-194-S3.PDF]

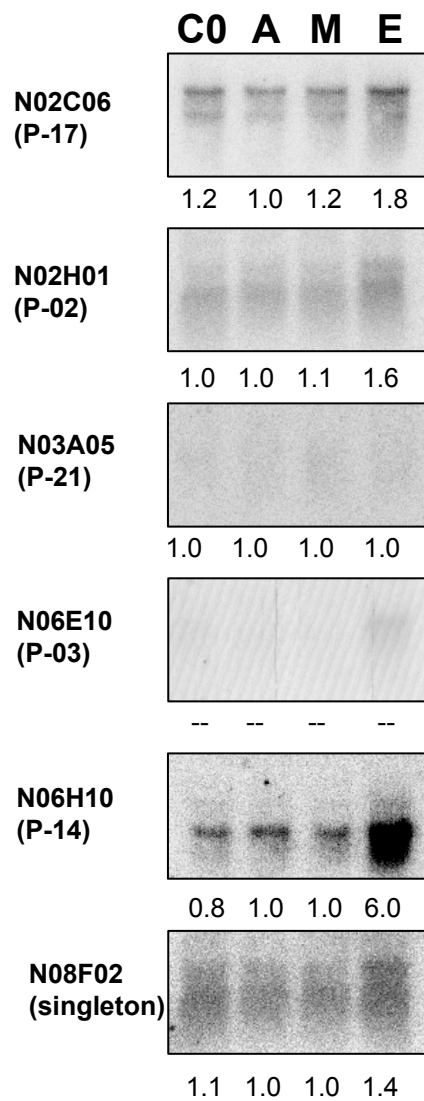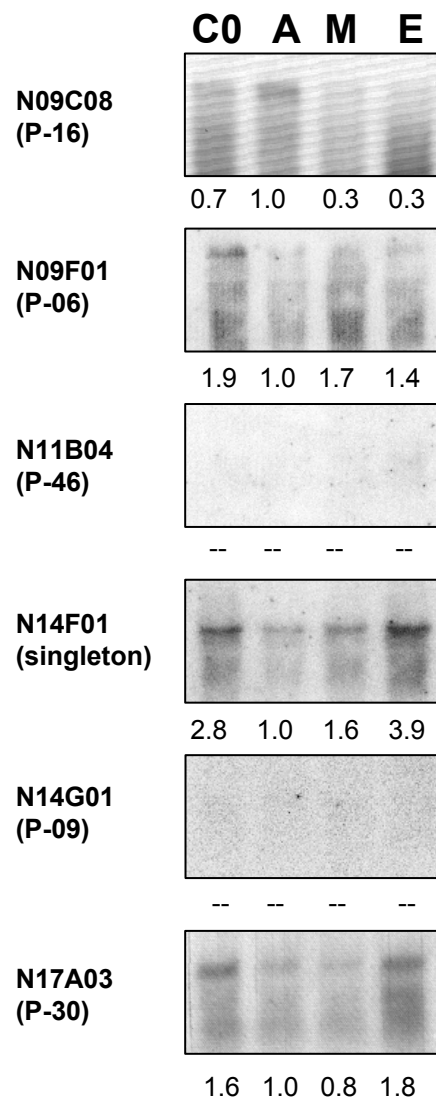

Supplement: Additional file 4 — Northern blot analysis of clones belonging to RindPdigS in C. sinensis Navelate fruit's peel after ethylene or 1-MCP pretreatment. Tissue samples were analysed before any treatment (C0) or after 16 h of treatment with air (A), 500 ppb xof 1-MCP (M), or 10 ppm of ethylene (E). In brackets it is indicated whether the clone is a singleton or the cluster it belongs to. Hybridization with the C. sinensis 26 S rDNA is shown in the bottom panel. Normalization of hybridization signals was carried out with respect to the hybridization signal of the C. sinensis rDNA. Values below the panels show the relative quantification of the corresponding hybridization signal referred to the value of the sample E. Those hybridization signals lower than two fold the background intensity were not assessed (-). [file 1471-2229-10-194-S4.PDF]
